# Effect of age and stereopsis on a multiple-object tracking task

**DOI:** 10.1371/journal.pone.0188373

**Published:** 2017-12-15

**Authors:** Marjolaine Plourde, Marie-Eve Corbeil, Jocelyn Faubert

**Affiliations:** 1 Visual Psychophysics and Perception Laboratory, School of Optometry, University of Montreal, Montreal, Quebec, Canada; 2 School of Optometry, University of Montreal, Montreal, Quebec, Canada; Universidad de Chile, CHILE

## Abstract

3D vision develops during childhood and tends to diminish after 65 years of age. It is still relatively unknown how stereopsis is used in more complex/ecological contexts such as when walking about in crowds where objects are in motion and occlusions occur. One task that shares characteristics with the requirements for processing dynamic crowds is the multiple object-tracking task (MOT). In the present study we evaluated the impact of stereopsis on a MOT task as a function of age. A total of 60 observers consisting of three groups of 20 subjects (7–12 years old, 18–40 years old and 65 years and older) completed the task in both conditions (with and without stereoscopic effects). The adult group obtained the better scores, followed by the children and the older adult group. The performance difference between the stereoscopic and non-stereoscopic conditions was significant and similar for the adults and the children but was non significant for the older observers. These results show that stereopsis helps children and adults accomplish a MOT task, but has no impact on older adults’ performances. The present results have implications as to how populations differ in their efficiency of using stereoscopic cues for disambiguating complex dynamic scenes.

## Introduction

The three-dimensional sensation obtained when observing the real world is in great part provided by the fact that both eyes have a slightly different perspective of the world. This slightly different view from each eye (disparity) is used by the brain to generate the strong depth sensation we observe, known as stereopsis. Although the role of stereopsis for generating depth perception is well known and was beautifully demonstrated in the seminal studies by Julesz, the emphasis has been primarily on static cues [1]. Yet our world is in motion and the role of stereoscopic mechanisms for perceiving depth for naturally occurring dynamic scenes is still relatively unknown. However, when we assess binocular depth perception in vision clinics, we only use static measures that require fine spatial resolution. Recent studies have implied that stereopsis can play a facilitation role in dynamic conditions such as when someone tracks multiple moving objects [2]. In fact studies have shown that speed thresholds in a multiple object tracking task (MOT) can be improved up to 50% [3] and that when objects occlude in the scene stereoscopic cues can improve speed thresholds by a factor of 3 [4]. Therefore, stereopsis can play a significant role in dynamic stimuli yet we have no knowledge at present on how age differences influence this capacity.

Stereopsis is not stable throughout life. A certain level of stereopsis can be measured in infants as early as 4 months of age [5–7]. Binocular vision then matures during childhood and the measured stereoacuity reaches the adult level between 4 years of age and up to 9 or 14 years of age, depending on the test used [7–9]. Leat & al [9] obtained adult level stereoacuity at 7 years of age with the *Frisby Test* and the *Randot Stereotest*^*®*^, two stereotests widely used by eye care professionals. Unfortunately, stereopsis is also affected by aging, and tends to deteriorate after 65 years of age, even with healthy, nonpathological, aging eyes [10–12]. Normal aging not only diminishes stereopsis, but also affects the processing of other second order stimuli [13]. The solicitation of these more complex neuronal processes further demonstrates alterations of visual functions with age [14].

In this study, we used the NeuroTracker^TM^, a three-dimensional multiple object tracking (3D-MOT) task developed by Jocelyn Faubert of University of Montreal. A classic 3D-MOT training includes 4 characteristics: MOT, a large visual field, speed thresholds and stereoscopy [3]. Several training sessions in these conditions improve the performances of almost any populations, from professional athletes to healthy elderly [3, 15]. In addition to reflecting a sport environment, 3D-MOT recreates a dynamic requirements similar to our everyday environment. With a paediatric population, the task parameters have to be adjusted: younger children can follow fewer targets compared to adults. Trick, Jaspers-Fayer & Sethi [16] measured lower performances in children up to 19 years of age. Decreased performances also occur with aging, and seniors have more difficulty following more then 3 targets. When speed threshold is used, older adults following 3 targets have similar results as young adults following 4 targets [17]. Knowing that stereoscopic cues improve the performances of adults on a MOT task and that children and older observers might not have complete stereopsis like adults, it is interesting to evaluate how stereopsis helps younger children and older adults accomplish this task. Furthermore, it has been demonstrated that aging reduces speed thresholds for the 3D-MOT task [15], yet it remains unresolved to what extent the decreased performance of the older adults is due solely to a reduction of higher-level cognitive functions solicited by the MOT component of the task or is compounded by the reduced effectiveness of the stereoscopy gain normally observed when performing this task in young healthy adults.

The goal of this study was to assess the impact of age and stereoscopy on a MOT task, by using the portable version of the NeuroTracker^TM^. We made the hypothesis that the adult group would have the best average results, and would benefit the most from the addition of stereoscopic effect. We also hypothesised that the reduced performances of the older observers would be the result of both a reduction of cognitive skills required to track multiple moving objects in addition to a reduced benefit of the stereoscopic cues used to disambiguate the elements during the task.

## Methods

### Participants

Three groups of 20 subjects were recruited: children (7–12 years old), adults (18–40 years old) and older adults (≥65 years old). The subjects were recruited in different areas of Montreal, through 3 different optometry clinics. Eleven subjects from the kids group were recruited at an elementary school in Laval, after a visual screening. All participants underwent a comprehensive eye exam or vision screening in the previous year. The vision screening was done by optometrists and included an evaluation of the visual acuity, binocular vision, refraction and eye health. They all had monocular distance visual acuity of 20/25 or better and monocular near visual acuity of 0,37M. They had to correctly identify the 6 shapes of the *Randot Stereotest*^*®*^, and their stereoacuity measured with the circles was recorded. Participants were excluded if they had a diagnosis of development disorder or attention deficit, or if they presented an ocular pathology. Participants were also excluded if they had already been trained with 3D-MOT. All participants agreed to this study and completed the informed consent form before the beginning of the session. This research was approved by the ethic committee CERES (Comité d’éthique de la recherché en santé) of University of Montreal.

### Stimuli and procedure

**Fig 1:**
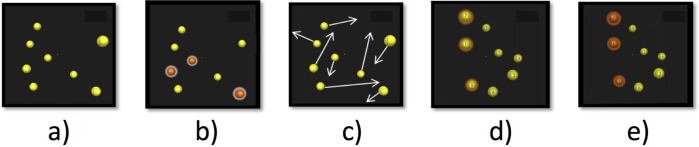
**Five steps of the 3D-MOT task** (a) presentation phase where 8 spheres are shown in a 3D volume space, (b) indexing phase where 3 spheres (targets) change colour (red) and are highlighted (hallo) for 1 second, (c) movement phase where the targets indexed in stage b return to their original form and colour and all spheres move for 6 seconds crisscrossing and bouncing off of each other and the virtual 3D volume cube walls that are not otherwise visible, (d) identification phase where the spheres come to a halt and the observer has to identify the 3 spheres originally indexed in phase (b). The spheres are individually tagged with a number so the observer can give the number corresponding to the original targets, and (e) feedback phase where the subject is given information on the correct targets [19].

The 3D-MOT used in this research consisted of 8 spheres moving in a virtual cube (Fig 1). At first, the 8 spheres were yellow (presentation phase, Fig 1A), and then three of them turned red for 1 second (indexing phase, Fig 1B). All 8 spheres returned to their original yellow color and began to move in all directions (X, Y and Z) in a virtual 3D cube (movement phase, Fig 1C). They bumped and occluded each other or bounced off the walls of the cube. After 6 seconds, the spheres stopped and the subject had to identify the three targets selected at the beginning of the trial (identification phase, Fig 1D). Finally, a feedback was given to the subject by highlighting the three initial targets (Fig 1E). The thresholds were obtained using a one up one down staircase procedure [18]. The trial always started at an arbitrary speed value of 1 (1 NeuroTracker unit represents 68 cm/s in virtual speed); if the subject correctly pointed out the 3 targets, the speed increased by 0.05 log units; if he made a mistake, it slowed down by the same proportion, resulting in a threshold criterion of 50%. The staircase was interrupted after twenty inversions and the threshold was calculated by the mean speed of the last four inversions.

For this research, two conditions were tested (with and without stereopsis), and 2 thresholds were obtained for each condition, by 2 separate staircase procedures. The mean of these two staircases was considered as the subject’s performance for this condition. The total duration of the session was approximately 25 minutes. The order of the two conditions was counterbalanced between subjects to cancel the effect of training. The number of targets, three, was chosen to make sure that all participants could perform the task without loosing interest during the session. A short presentation video explained the procedure before the session. The stereoscopic effect was created by anaglyph and subjects wore red-blue glasses. The stereopsis condition showed a disparity range from 0 to 150 seconds of arc depending on the actual sphere position in the virtual cube. The portable version of the 3D-MOT was tested by using a tablet to display the 3D-MOT, at a fixed 40 cm viewing distance. The tablet used was a *ASUS* Transformer Book T100TAM, a 10.1” Windows tablet (1366 x 768 pixels).

## Results

All 60 subjects were able to complete the task on the portable version of the 3D-MOT. The children group obtained a mean stereoacuity (measured with the circles of the *Randot Stereotest*^*®*^) of 24,50 ± 5,60 arc seconds, the adult group 22,50 ± 3,44 arc seconds and the elderly group 94,00 ± 86,84 arc seconds. The mean age for the children was 7,95 ± 1,36 years (range: 7–12), adults 26,20 ± 3,49 years (range: 22–37) and older adults 69,05 ± 4,57 years (range: 65–77).

**Fig 2:**
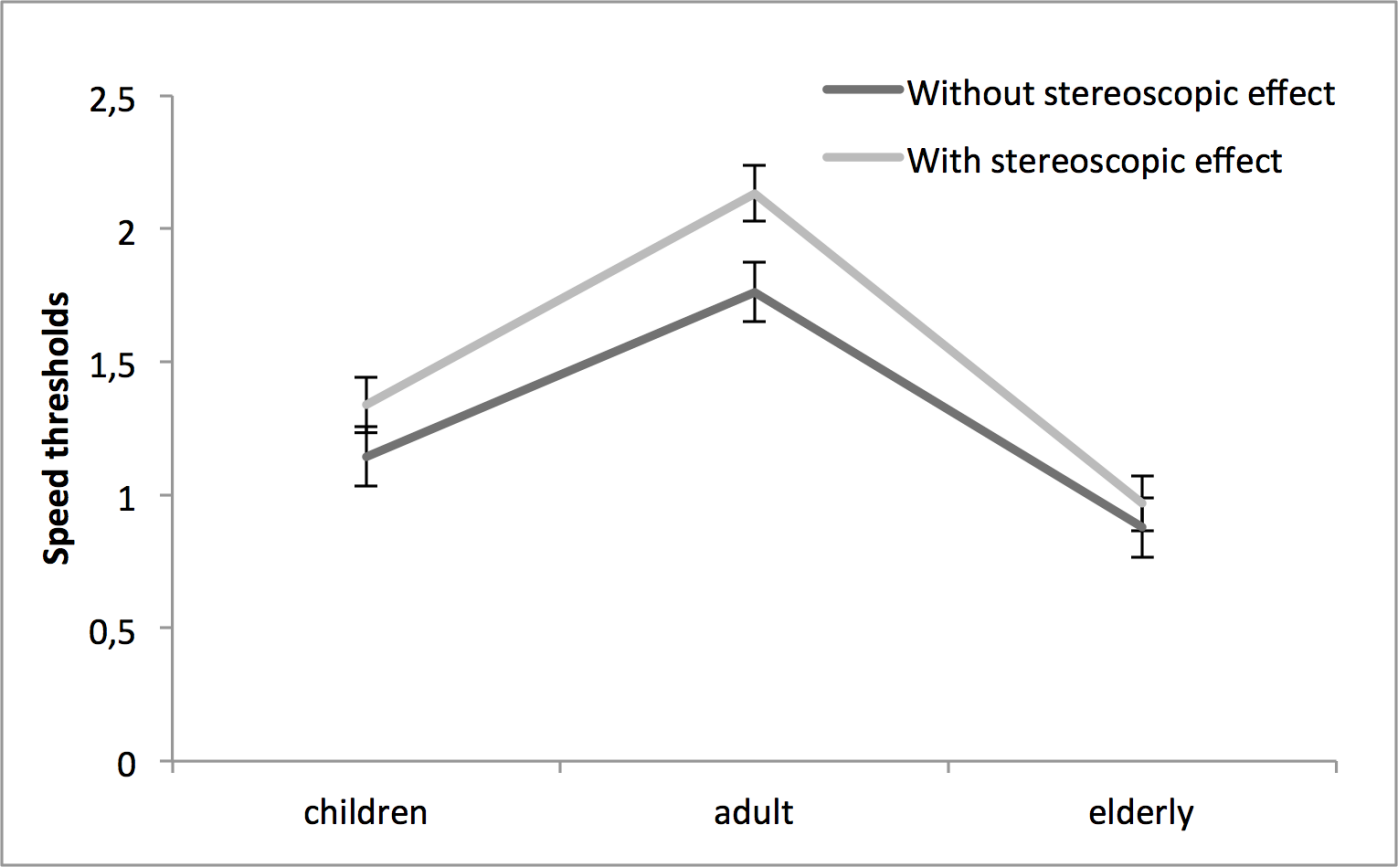
Scores with standard errors for each condition and group.

The average score for each condition and group is shown in Fig 2. A mixed ANOVA confirms the significant impact of age (*F*(2,57) = 27,063; *p*<0,01; η_p_^2^ = .487), stereoscopic effect (*F*(1,57) = 24,259; *p*<0,01; η_p_^2^ = .299), and age by stereopsis interaction (*F*(2,57) = 3,408; *p*<0,05; η_p_^2^ = .107). A post-hoc analyse (Fisher LSD) shows a significant difference between the adult and children group (p<0,01), between the adult and elderly group (p<0,01) and finally between the children and elderly group (p<0,05). The average performances (mean of both conditions) for the adult group are higher then those of the children group, which are higher then those of the elderly group.

**Fig 3:**
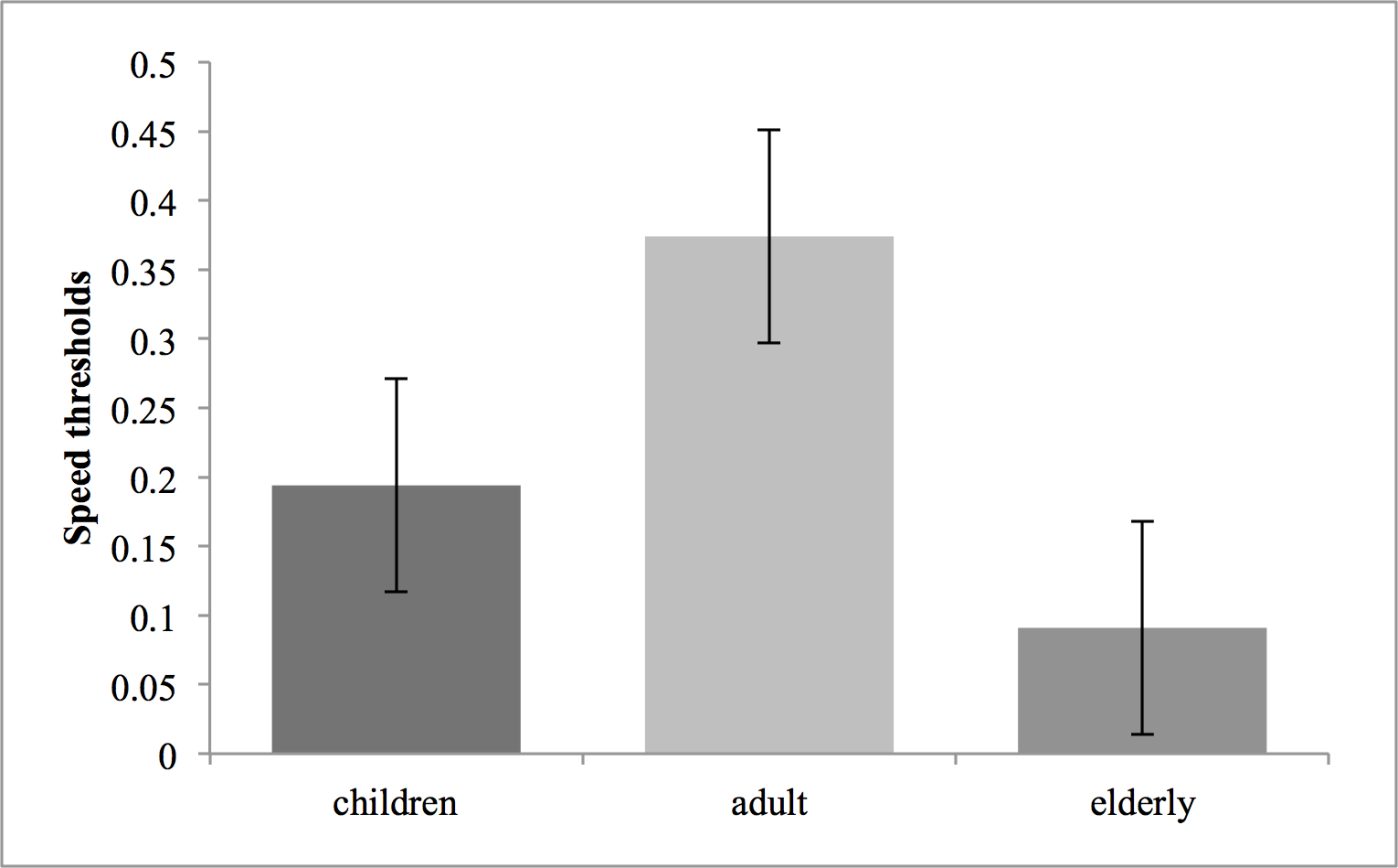
Score differences between both conditions (stereopsis advantage) with standard errors for each group.

The difference scores between the stereoscopic and non-stereoscopic conditions are also different between groups. The adult group had a difference score of 0,374 (± 0,077), versus 0,194 (± 0,077) for the children group and 0,091 (± 0,077) for the elderly group. These score differences, which we will call the stereopsis advantage, are shown in Fig 3. A one-way ANOVA considering the stereopsis advantage is statistically significant F (2,59): 3,453 (p<0,04). A post-hoc Fisher LSD shows a significant difference between the adult and elderly group only (p<0,05): the performances improvement associated with stereopsis is diminished in elderlies, compared to adults. Also, *t* tests with Bonferroni correction confirm the significant differences between both conditions for the adult group (*t*(19) = 3,875; p = 0,001) and the children group (*t*(19) = 2,938; p = 0,008). This difference is non-significant for the elderly group. Therefore, the stereopsis advantage is greater in adults and similar with kids, but is almost zero for older adults.

When trying to correlate the 3D-MOT scores to the *Randot Stereotest*^*®*^ stereoacuities, only the older adult group can be considered. Indeed, a majority of the adult and children group have reached the maximum stereoacuity of 20 seconds of arc measurable with this test. The *Randot Stereotest*^*®*^ stereoacuities for the older adult group are not significantly correlated to the 3D-MOT scores (r = -0,112 ± 0,638), which suggest that these two tasks do not measure the same stereoscopic processing.

## Discussion

To our knowledge, this research is the first to evaluate stereopsis influence on MOT performances in 3 age population groups from childhood to older age. The average MOT results (mean of both conditions) confirm the progression of the performances in a MOT task in school-aged children. As argued by Trick, Jaspers-Fayer & Sethi [16] and Koldewyn & al [20], a significant difference between the adult and children mean performances could be related to an evolution of attention, working memory and visual information processing speed, functions involved in MOT. The lower mean performances in the older adult group, which support the results of several other studies [15, 21, 22], may indicate deterioration of these same functions. However, the present results clearly demonstrate that the aging effect observed in the 3D-MOT scores also emanates from a loss of the stereoscopic advantage that the other groups younger groups benefit from.

The comparison between scores with and without stereoscopic effect allows us to directly evaluate the stereopsis advantage. The non-significant impact of stereoscopic effect on the elderly’s results does not directly relate to their lower depth sensitivity recorded at the *Randot Stereotest*^*®*^, as the results of the Randot and 3D-MOT are not correlated. The lack of improvement by the addition of stereoscopic effect could be related to a different use of stereoscopic information that also changes with age. As we know, normal aging affects perception of second order stimuli to a greater extent than first order stimuli [14]. It was suggested that second order stimuli (stereopsis is also a second order process) require more complex networks for efficient processing and that these are particularly affected by the normal aging process [13]. The lack of stereopsis advantage in the elderly group may be due to this alteration of second order stimuli processing, resulting in a reduced ability to efficiently use stereoscopic cues in a dynamic stimulus such as in 3D-MOT.

The higher performances in stereoscopic condition were anticipated for the adult and children group. O'Connor & al [23] and Alramis & al [24] demonstrated the advantage of binocularity in fine motor tasks, for adults and children respectively. What is more surprising here is the absence of significant difference in the stereopsis advantage between the adult and children group. Indeed, several other teams showed a progression in stereopsis from childhood to teenage years. Giaschi & al [7] recorded lower performances up to 14 years old with fine disparity, using their computerized stereotest. With the *Randot Stereotest*^*®*^, we obtained similar stereoacuities for children and adult, confirming the results of Leat & al [9]. The 3D-MOT task used in this study shows disparity from 0 to 150 seconds of arc. We hypothesised that both children and older adults may have a reduced benefit from stereopsis but for different reasons. The children because the stereopsis processing mechanisms may not be yet mature and the older observers because the efficiency, given that it is a second order process, may be reduced by aging. Our results only support the latter. Nevertheless, a trend is still visible between the children and adult stereopsis advantage: the development process may not be totally complete in 7–12 years old children. With even younger subjects the stereopsis advantage may be significantly different from young adults.

Another explanation for the similar stereopsis advantage measured in the children and adult group could be the different nature of the 3D used in a 3D-MOT task, which represents dynamic stereopsis. This type of stereopsis might not develop at the same rate as static stereopsis. To our knowledge, no other study has evaluated the development of dynamic stereopsis in children. This stereopsis may have reached adult levels at 7 to 12 years of age and be diminished by 65 years old. This type of 3D might better reflect our everyday use of stereopsis. A weak, even null, correlation between dynamic and static stereoacuity has been documented before [25, 26]. More recently, Tidbury & al [27] showed that dynamic disparities were more effective for generating depth perception than static ones.

One limitation of the present study is the more compact instrumentation used, compared to previous studies where a much larger virtual environment was used [3, 15, 28]. The smaller screen limits the visual field and the range of disparity that can be displayed. Using a wider screen, the stereoscopic advantage for every group could possibly be higher. In fact, with a larger and more immersive environment, the addition of strereoscopic effect improved the scores of adults by 50%, against 21% for our adult group [28]. Using a wider screen could reveal a significant impact of stereopsis on the senior group results, by showing even coarser disparities. However, the portable version is usable with any age group, and may become a tool for home training or clinical evaluation, in assessing a more dynamic use of stereopsis. Further studies are needed to establish the parameters of such clinical or training tools.

## Conclusion

The portable version of the NeuroTracker^TM^ is easily usable with older observers as well as with kids. The adults perform better then children and older adults, and adults and children benefit equally from the addition of stereoscopy for this MOT task.

## Supporting information

S1 File Data. (XLSX)
